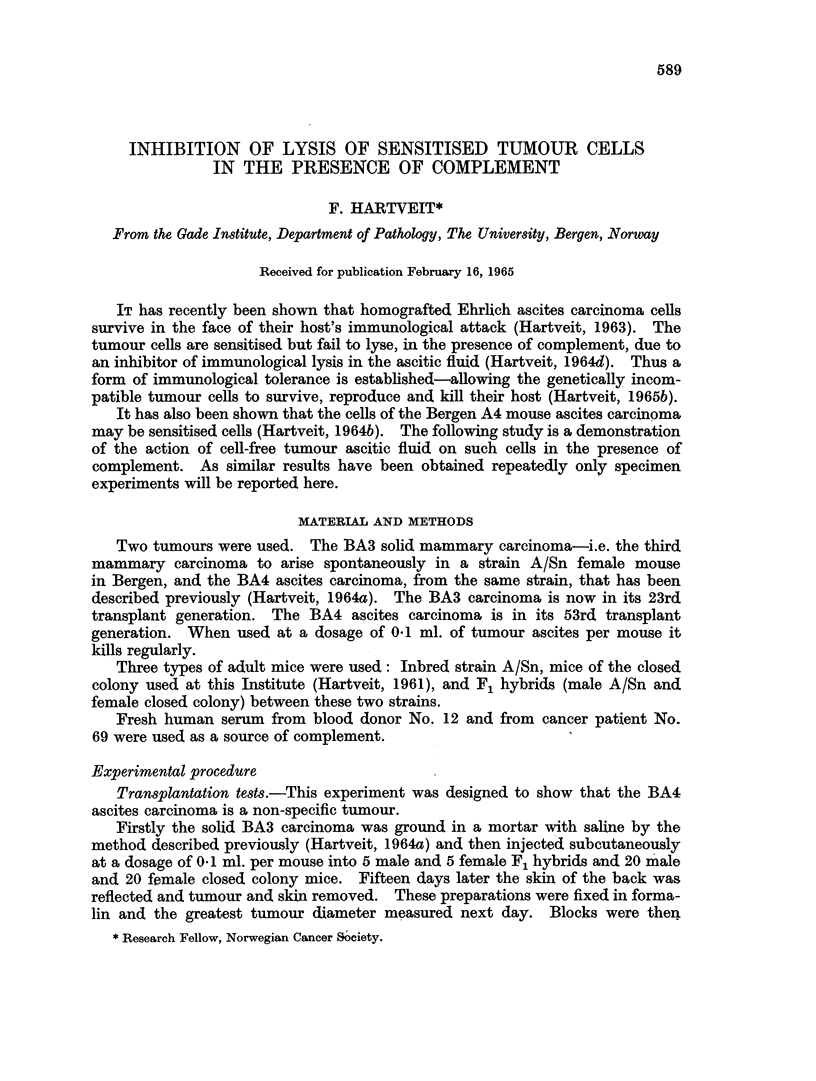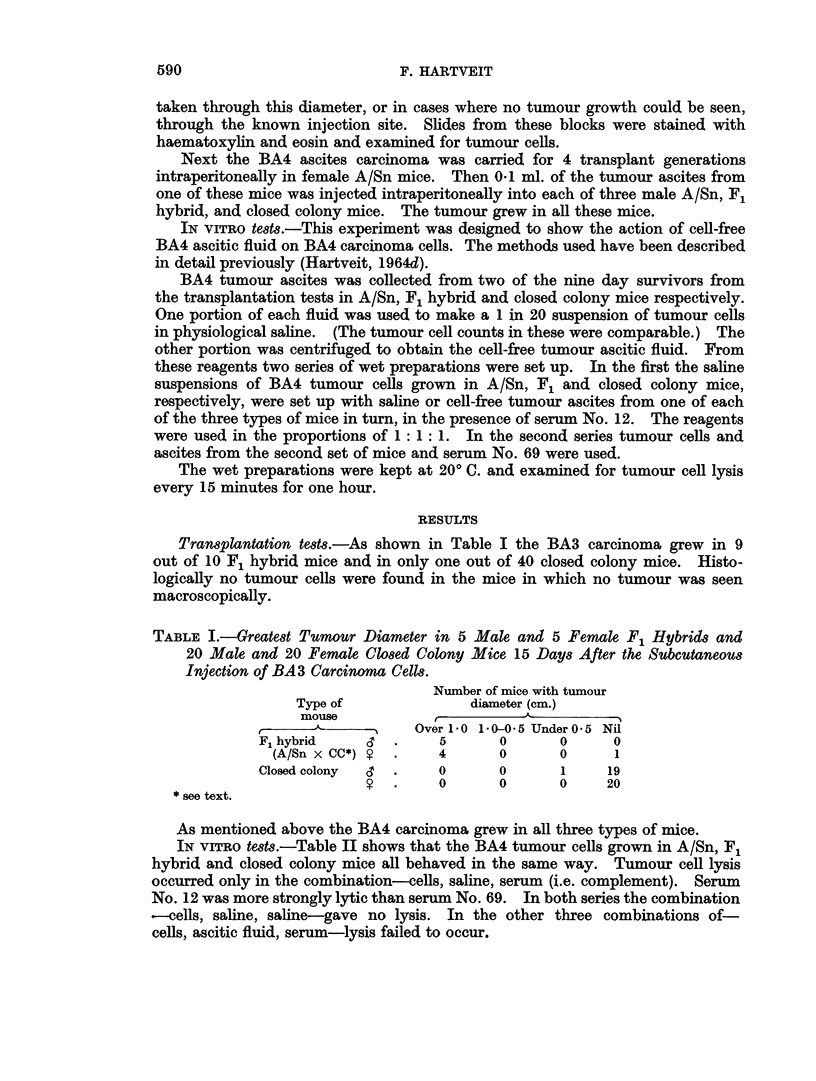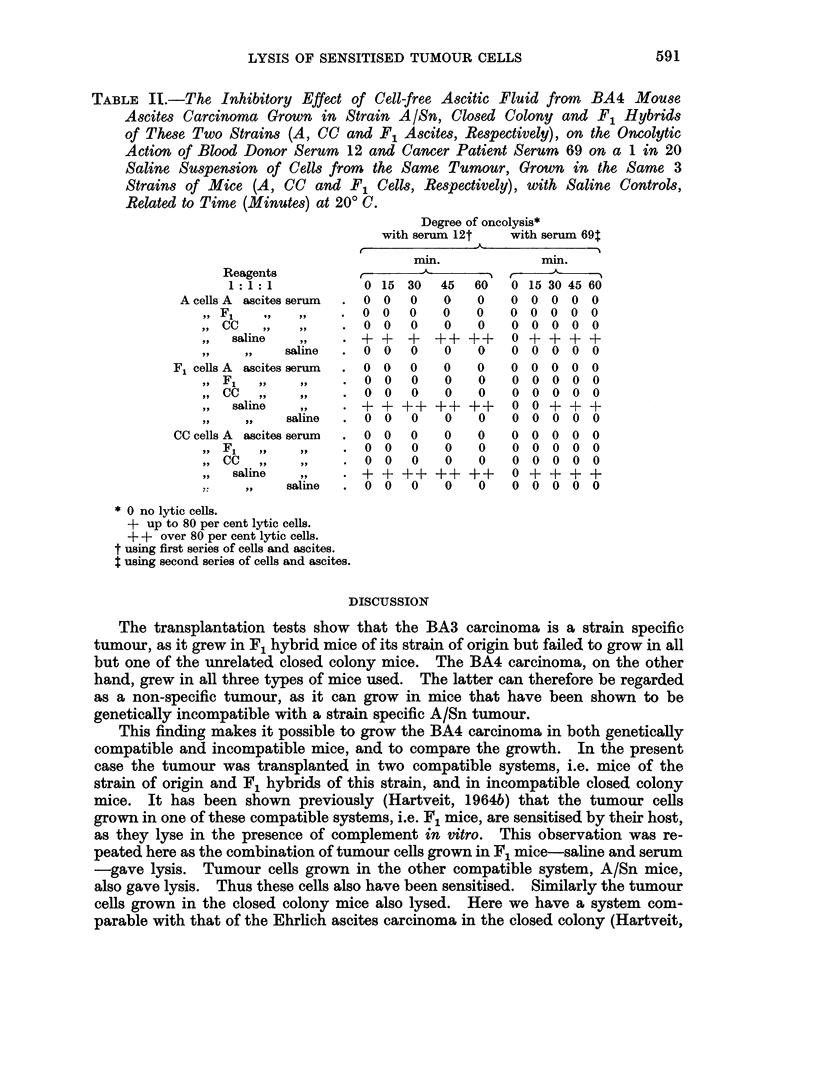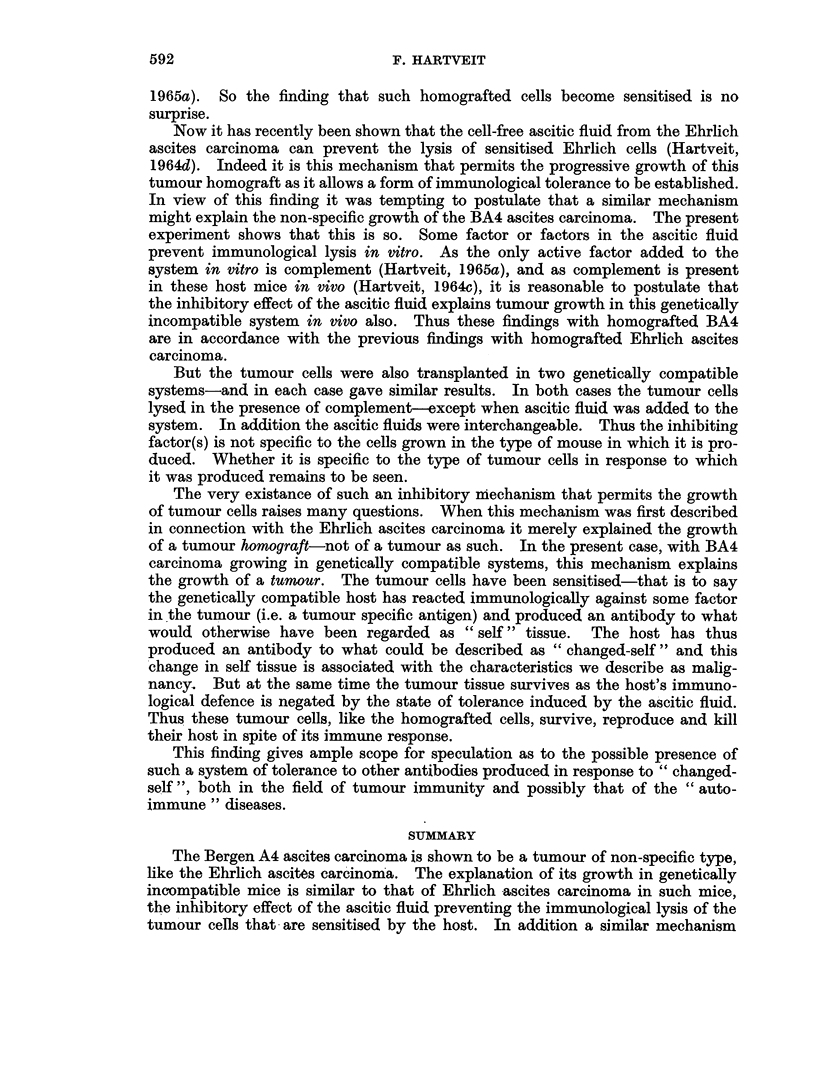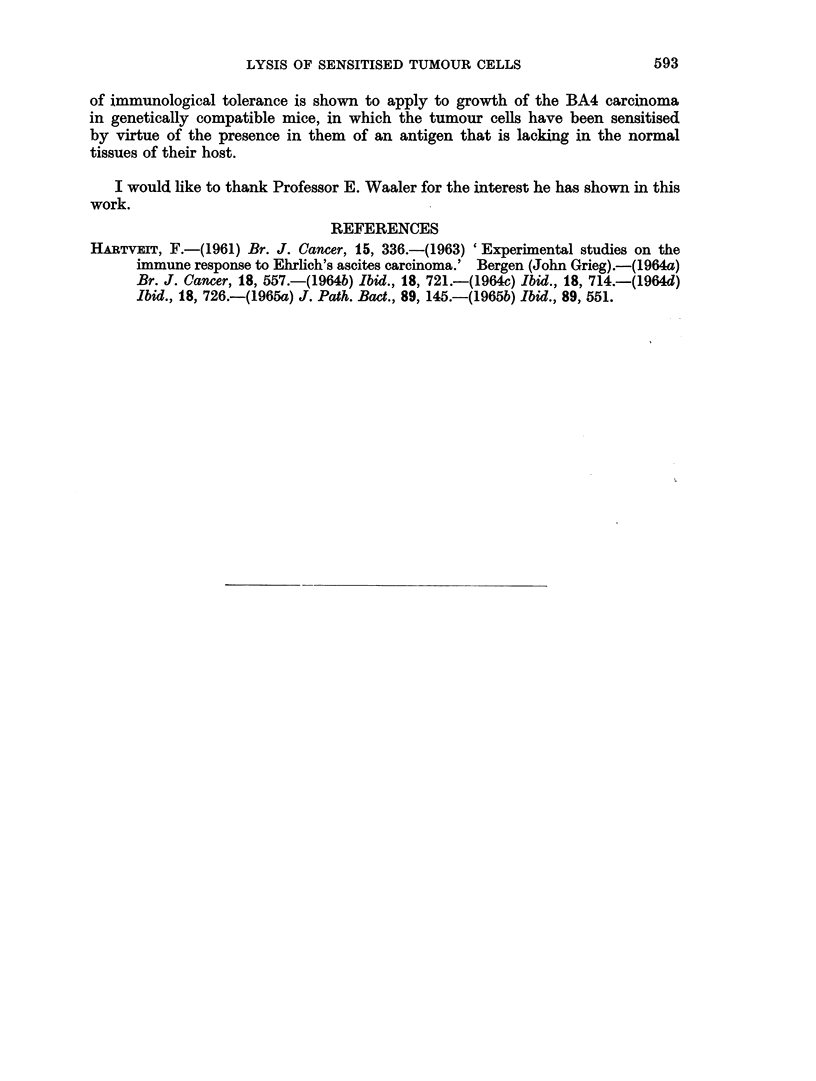# Inhibition of lysis of sensitised tumour cells in the presence of complement.

**DOI:** 10.1038/bjc.1965.73

**Published:** 1965-09

**Authors:** F. Hartveit


### INHIBITION OF LYSIS OF SENSITISED TUMOUR CELLS IN THE PRESENCE OF COMPLEMENT

F. HARTVEIT*

*From the Gade Institute, Department of Pathology, The University, Bergen, Norway*

Received for publication February 16, 1965

IT has recently been shown that homografted Ehrlich ascites carcinoma cells survive in the face of their host's immunological attack (Hartveit, 1963). The tumour cells are sensitised but fail to lyse, in the presence of complement, due to an inhibitor of immunological lysis in the ascitic fluid (Hartveit, 1964*d*). Thus a form of immunological tolerance is established—allowing the genetically incompatible tumour cells to survive, reproduce and kill their host (Hartveit, 1965*b*).

It has also been shown that the cells of the Bergen A4 mouse ascites carcinoma may be sensitised cells (Hartveit, 1964*b*). The following study is a demonstration of the action of cell-free tumour ascitic fluid on such cells in the presence of complement. As similar results have been obtained repeatedly only specimen experiments will be reported here.

### MATERIAL AND METHODS

Two tumours were used. The BA3 solid mammary carcinoma—i.e. the third mammary carcinoma to arise spontaneously in a strain A/Sn female mouse in Bergen, and the BA4 ascites carcinoma, from the same strain, that has been described previously (Hartveit, 1964*a*). The BA3 carcinoma is now in its 23rd transplant generation. The BA4 ascites carcinoma is in its 53rd transplant generation. When used at a dosage of 0·1 ml. of tumour ascites per mouse it kills regularly.

Three types of adult mice were used: Inbred strain A/Sn, mice of the closed colony used at this Institute (Hartveit, 1961), and $F_1$ hybrids (male A/Sn and female closed colony) between these two strains.

Fresh human serum from blood donor No. 12 and from cancer patient No. 69 were used as a source of complement.

### *Experimental procedure*

*Transplantation tests.*—This experiment was designed to show that the BA4 ascites carcinoma is a non-specific tumour.

Firstly the solid BA3 carcinoma was ground in a mortar with saline by the method described previously (Hartveit, 1964*a*) and then injected subcutaneously at a dosage of 0·1 ml. per mouse into 5 male and 5 female $F_1$ hybrids and 20 male and 20 female closed colony mice. Fifteen days later the skin of the back was reflected and tumour and skin removed. These preparations were fixed in formalin and the greatest tumour diameter measured next day. Blocks were then

* Research Fellow, Norwegian Cancer Society.

taken through this diameter, or in cases where no tumour growth could be seen, through the known injection site. Slides from these blocks were stained with haematoxylin and eosin and examined for tumour cells.

Next the BA4 ascites carcinoma was carried for 4 transplant generations intraperitoneally in female A/Sn mice. Then 0·1 ml. of the tumour ascites from one of these mice was injected intraperitoneally into each of three male A/Sn, $F_1$ hybrid, and closed colony mice. The tumour grew in all these mice.

IN VITRO *tests.*—This experiment was designed to show the action of cell-free BA4 ascitic fluid on BA4 carcinoma cells. The methods used have been described in detail previously (Hartveit, 1964*d*).

BA4 tumour ascites was collected from two of the nine day survivors from the transplantation tests in A/Sn, $F_1$ hybrid and closed colony mice respectively. One portion of each fluid was used to make a 1 in 20 suspension of tumour cells in physiological saline. (The tumour cell counts in these were comparable.) The other portion was centrifuged to obtain the cell-free tumour ascitic fluid. From these reagents two series of wet preparations were set up. In the first the saline suspensions of BA4 tumour cells grown in A/Sn, $F_1$ and closed colony mice, respectively, were set up with saline or cell-free tumour ascites from one of each of the three types of mice in turn, in the presence of serum No. 12. The reagents were used in the proportions of 1 : 1 : 1. In the second series tumour cells and ascites from the second set of mice and serum No. 69 were used.

The wet preparations were kept at 20° C. and examined for tumour cell lysis every 15 minutes for one hour.

### RESULTS

*Transplantation tests.*—As shown in Table I the BA3 carcinoma grew in 9 out of 10 $F_1$ hybrid mice and in only one out of 40 closed colony mice. Histologically no tumour cells were found in the mice in which no tumour was seen macroscopically.

TABLE I.—*Greatest Tumour Diameter in* 5 *Male and* 5 *Female* $F_1$ *Hybrids and* 20 *Male and* 20 *Female Closed Colony Mice* 15 *Days After the Subcutaneous Injection of BA*3 *Carcinoma Cells.*

| Type of mouse | | Number of mice with tumour diameter (cm.) | | | |
|---|---|---|---|---|---|
| | | Over 1·0 | 1·0–0·5 | Under 0·5 | Nil |
| $F_1$ hybrid | ♂ | 5 | 0 | 0 | 0 |
| (A/Sn × CC*) | ♀ | 4 | 0 | 0 | 1 |
| Closed colony | ♂ | 0 | 0 | 1 | 19 |
| | ♀ | 0 | 0 | 0 | 20 |

* see text.

As mentioned above the BA4 carcinoma grew in all three types of mice.

IN VITRO *tests.*—Table II shows that the BA4 tumour cells grown in A/Sn, $F_1$ hybrid and closed colony mice all behaved in the same way. Tumour cell lysis occurred only in the combination—cells, saline, serum (i.e. complement). Serum No. 12 was more strongly lytic than serum No. 69. In both series the combination —cells, saline, saline—gave no lysis. In the other three combinations of—cells, ascitic fluid, serum—lysis failed to occur.

TABLE II.—*The Inhibitory Effect of Cell-free Ascitic Fluid from BA4 Mouse Ascites Carcinoma Grown in Strain A/Sn, Closed Colony and $F_1$ Hybrids of These Two Strains (A, CC and $F_1$ Ascites, Respectively), on the Oncolytic Action of Blood Donor Serum* 12 *and Cancer Patient Serum* 69 *on a* 1 *in* 20 *Saline Suspension of Cells from the Same Tumour, Grown in the Same* 3 *Strains of Mice (A, CC and $F_1$ Cells, Respectively), with Saline Controls, Related to Time (Minutes) at* 20° *C.*

| | Degree of oncolysis* with serum 12† | | | | | with serum 69‡ | | | | |
|---|---|---|---|---|---|---|---|---|---|---|
| | min. | | | | | min. | | | | |
| Reagents 1 : 1 : 1 | 0 | 15 | 30 | 45 | 60 | 0 | 15 | 30 | 45 | 60 |
| A cells A ascites serum | 0 | 0 | 0 | 0 | 0 | 0 | 0 | 0 | 0 | 0 |
| ,, $F_1$ ,, ,, | 0 | 0 | 0 | 0 | 0 | 0 | 0 | 0 | 0 | 0 |
| ,, CC ,, ,, | 0 | 0 | 0 | 0 | 0 | 0 | 0 | 0 | 0 | 0 |
| ,, saline ,, | + | + | + | ++ | ++ | 0 | + | + | + | + |
| ,, ,, saline | 0 | 0 | 0 | 0 | 0 | 0 | 0 | 0 | 0 | 0 |
| $F_1$ cells A ascites serum | 0 | 0 | 0 | 0 | 0 | 0 | 0 | 0 | 0 | 0 |
| ,, $F_1$ ,, ,, | 0 | 0 | 0 | 0 | 0 | 0 | 0 | 0 | 0 | 0 |
| ,, CC ,, ,, | 0 | 0 | 0 | 0 | 0 | 0 | 0 | 0 | 0 | 0 |
| ,, saline ,, | + | + | ++ | ++ | ++ | 0 | 0 | + | + | + |
| ,, ,, saline | 0 | 0 | 0 | 0 | 0 | 0 | 0 | 0 | 0 | 0 |
| CC cells A ascites serum | 0 | 0 | 0 | 0 | 0 | 0 | 0 | 0 | 0 | 0 |
| ,, $F_1$ ,, ,, | 0 | 0 | 0 | 0 | 0 | 0 | 0 | 0 | 0 | 0 |
| ,, CC ,, ,, | 0 | 0 | 0 | 0 | 0 | 0 | 0 | 0 | 0 | 0 |
| ,, saline ,, | + | + | ++ | ++ | ++ | 0 | + | + | + | + |
| ,, ,, saline | 0 | 0 | 0 | 0 | 0 | 0 | 0 | 0 | 0 | 0 |

* 0 no lytic cells.
\+ up to 80 per cent lytic cells.
++ over 80 per cent lytic cells.
† using first series of cells and ascites.
‡ using second series of cells and ascites.

### DISCUSSION

The transplantation tests show that the BA3 carcinoma is a strain specific tumour, as it grew in $F_1$ hybrid mice of its strain of origin but failed to grow in all but one of the unrelated closed colony mice. The BA4 carcinoma, on the other hand, grew in all three types of mice used. The latter can therefore be regarded as a non-specific tumour, as it can grow in mice that have been shown to be genetically incompatible with a strain specific A/Sn tumour.

This finding makes it possible to grow the BA4 carcinoma in both genetically compatible and incompatible mice, and to compare the growth. In the present case the tumour was transplanted in two compatible systems, i.e. mice of the strain of origin and $F_1$ hybrids of this strain, and in incompatible closed colony mice. It has been shown previously (Hartveit, 1964*b*) that the tumour cells grown in one of these compatible systems, i.e. $F_1$ mice, are sensitised by their host, as they lyse in the presence of complement *in vitro*. This observation was repeated here as the combination of tumour cells grown in $F_1$ mice—saline and serum—gave lysis. Tumour cells grown in the other compatible system, A/Sn mice, also gave lysis. Thus these cells also have been sensitised. Similarly the tumour cells grown in the closed colony mice also lysed. Here we have a system comparable with that of the Ehrlich ascites carcinoma in the closed colony (Hartveit,

1965*a*). So the finding that such homografted cells become sensitised is no surprise.

Now it has recently been shown that the cell-free ascitic fluid from the Ehrlich ascites carcinoma can prevent the lysis of sensitised Ehrlich cells (Hartveit, 1964*d*). Indeed it is this mechanism that permits the progressive growth of this tumour homograft as it allows a form of immunological tolerance to be established. In view of this finding it was tempting to postulate that a similar mechanism might explain the non-specific growth of the BA4 ascites carcinoma. The present experiment shows that this is so. Some factor or factors in the ascitic fluid prevent immunological lysis *in vitro*. As the only active factor added to the system *in vitro* is complement (Hartveit, 1965*a*), and as complement is present in these host mice *in vivo* (Hartveit, 1964*c*), it is reasonable to postulate that the inhibitory effect of the ascitic fluid explains tumour growth in this genetically incompatible system *in vivo* also. Thus these findings with homografted BA4 are in accordance with the previous findings with homografted Ehrlich ascites carcinoma.

But the tumour cells were also transplanted in two genetically compatible systems—and in each case gave similar results. In both cases the tumour cells lysed in the presence of complement—except when ascitic fluid was added to the system. In addition the ascitic fluids were interchangeable. Thus the inhibiting factor(s) is not specific to the cells grown in the type of mouse in which it is produced. Whether it is specific to the type of tumour cells in response to which it was produced remains to be seen.

The very existance of such an inhibitory mechanism that permits the growth of tumour cells raises many questions. When this mechanism was first described in connection with the Ehrlich ascites carcinoma it merely explained the growth of a tumour *homograft*—not of a tumour as such. In the present case, with BA4 carcinoma growing in genetically compatible systems, this mechanism explains the growth of a *tumour*. The tumour cells have been sensitised—that is to say the genetically compatible host has reacted immunologically against some factor in the tumour (i.e. a tumour specific antigen) and produced an antibody to what would otherwise have been regarded as " self " tissue. The host has thus produced an antibody to what could be described as " changed-self " and this change in self tissue is associated with the characteristics we describe as malignancy. But at the same time the tumour tissue survives as the host's immunological defence is negated by the state of tolerance induced by the ascitic fluid. Thus these tumour cells, like the homografted cells, survive, reproduce and kill their host in spite of its immune response.

This finding gives ample scope for speculation as to the possible presence of such a system of tolerance to other antibodies produced in response to " changed-self ", both in the field of tumour immunity and possibly that of the " auto-immune " diseases.

### SUMMARY

The Bergen A4 ascites carcinoma is shown to be a tumour of non-specific type, like the Ehrlich ascites carcinoma. The explanation of its growth in genetically incompatible mice is similar to that of Ehrlich ascites carcinoma in such mice, the inhibitory effect of the ascitic fluid preventing the immunological lysis of the tumour cells that are sensitised by the host. In addition a similar mechanism of immunological tolerance is shown to apply to growth of the BA4 carcinoma in genetically compatible mice, in which the tumour cells have been sensitised by virtue of the presence in them of an antigen that is lacking in the normal tissues of their host.

I would like to thank Professor E. Waaler for the interest he has shown in this work.

### REFERENCES

HARTVEIT, F.—(1961) *Br. J. Cancer*, **15**, 336.—(1963) 'Experimental studies on the immune response to Ehrlich's ascites carcinoma.' Bergen (John Grieg).—(1964*a*) *Br. J. Cancer*, **18**, 557.—(1964*b*) *Ibid.*, **18**, 721.—(1964*c*) *Ibid.*, **18**, 714.—(1964*d*) *Ibid.*, **18**, 726.—(1965*a*) *J. Path. Bact.*, **89**, 145.—(1965*b*) *Ibid.*, **89**, 551.